# Lipopolysaccharides of *Brucella suis* S2 Impaired the Process of Decidualization in Early Pregnancy in Mice

**DOI:** 10.3390/toxins15110662

**Published:** 2023-11-16

**Authors:** Lanjie Lei, Xiangguo Wang, Jianpo Zhang, Jiaojiao Yin, Qin Xu, Ting Wang, Yaping Jin, Aihua Wang

**Affiliations:** 1 Key Laboratory of Animal Biotechnology of the Ministry of Agriculture and Rural Affairs, Northwest A&F University, Xianyang 712100, China; leilanjie1988@163.com (L.L.); xiangguo731@163.com (X.W.); zhjp821@126.com (J.Z.); yinjj198904@163.com (J.Y.); zh4913xuqin40467@126.com (Q.X.); wt187008490521@outlook.com (T.W.); yapingjin@163.com (Y.J.); 2 College of Veterinary Medicine, Northwest A&F University, Xianyang 712100, China

**Keywords:** *Brucella suis* S2, lipopolysaccharide, mice, pregnancy loss, decidualization

## Abstract

*Brucellosis* is a notorious zoonotic disease caused by *Brucella*, which can lead to reproductive diseases in humans and animals, such as infertility and abortion. Lipopolysaccharides (LPS) are the main virulence factor of *Brucella*. LPS derived from *Brucella* are different and non-classical and are less toxic and less active than LPS isolated from *E. coli.* However, the effects and possible mechanisms of *Brucella* LPS-caused pregnancy loss remain to be revealed. In the present study, we investigated the effects of *Brucella suis* S2 LPS on early pregnancy loss in mice. The results indicated that embryo implantation failure was induced by *Brucella* LPS treatment in a dose-dependent manner. The injection of *Brucella* LPS mainly resulted in fibrinolysis in the decidual area of the uterus on the 6th day post coition (dpc), infiltration of large granular cells among the decidual cells near the embryo on the 8th dpc, a large number of gaps in the decidual area, and cell necrosis around the embryo. In addition, the expression of Cyclin D3 mRNA in the uterus on the 7th and 8th dpc and IGFBP-1 mRNA and the progesterone receptor in the uterus on the 6th and 7th dpc were also inhibited. Moreover, the expression of decidualization marker Cyclin D3 and decidualization prolactin-associated protein (dPRP) in endometrial stromal cells were also inhibited by *Brucella* LPS treatment in vitro. In summary, *Brucella* LPS affect the process of endometrial decidualization in mice by affecting the structure of the decidua and the expression of decidual marker factors in endometrial stromal cells.

## 1. Introduction

Brucellosis is a zoonotic disease that is widely spread worldwide. *Brucella*, the causative agent of brucellosis, is a facultative intracellular Gram-negative bacteria [1]. *Brucella* mainly harms the reproductive systems of humans and animals, resulting in preterm birth, abortion, placenta retention, weak fetus, malformed fetus, stillbirth, and infertility caused by orchitis or epididymitis in male animals, etc., and it can also cause systemic illness and skin lesions in humans and requires months of treatment before it resolves [2].

Successful establishment of pregnancy depends on the ability of the blastocyst to adhere to the uterine luminal epithelium and the endometrium to grow and transform into a receptive state [3]. In rodents and primates, decidualization of uterine stromal cells is necessary for embryo implantation and is an important step in a successful pregnancy. Under the action of ovarian steroid hormones, fibroblasts in the uterine stroma surrounding the blastocyst undergo extensive proliferation and differentiation, and the extracellular stroma also begins to rebuild, completing endometrial decidualization [4]. The process of endometrial decidualization involves the interactions and regulation of adhesion molecules, growth factors, and other proteins [4].

Related studies have confirmed that the implantation of mouse embryos begins in the early morning on the 4th day post coition (dpc). Meanwhile, stromal cells around the blastocyst adhesion site begin to proliferate and differentiate extensively, forming a type of decidualized cell with multiploidy [5]. On the 5th and 6th dpc, stromal cells around the site of blastocyst attachment stop proliferating but continue to differentiate, gradually forming the primary decidual zone [5]. Subsequently, stromal cells adjacent to the primary decidual zone continue to proliferate and differentiate, forming polyploidy decidual cells and gradually forming the secondary decidual zone until the 8th dpc. Finally, the decidualized cells in the secondary decidua region gradually undergo apoptosis, which can enlarge the lacunae of the blastocyst attachment site and enable the development of the embryo [6]. 

Under physiological conditions, endometrial decidualization is mainly regulated by estrogen (E2) and progesterone (P4) secreted by the ovary. In vitro, E2 and P4 can be added to endometrial stromal cells isolated from the uteruses of mice on the 4th dpc to induce decidualization to establish a decidualization model [7]. Decidualized stromal cells of the endometrium mainly secrete prolactin (PRL) and insulin-like growth factor binding protein 1 (IGFBP-1) to provide nutritional support to the embryo, protect the zygote, and reduce local immune responses [8]. In addition, the expression of Cyclin D1, Cyclin E, and CDK4 during the proliferation of endometrial epithelial cells during decidualization is mainly regulated by E2 treatment [9]. Cyclin D3 is associated with the proliferation, differentiation, and polyploidy of decidualized stromal cells [6], and it is a key factor in the process of endometrial cell proliferation and decidua polyploidy [10,11]. 

Studies have reported that embryo attachment sites are highly sensitive to pro-inflammatory factors such as *Escherichia coli* LPS and inflammatory factors such as IFN, TNF, and IL-2 induced by *E. coli* LPS, and this can eventually lead to embryo absorption in the early pregnancy of mice [12]. In addition, these inflammatory factors also play important roles in the implantation of blastocysts into the endometrium [13]. The expression of the progesterone receptor in decidual cells can be down-regulated by *E. coli* LPS treatment [14,15]. Changes in IL-1 expression levels can also be induced by LPS during the “window of reception” stage in the embryo and endometrium, suggesting that LPS-induced inflammation can cause embryo loss by affecting the decidualization process [16].

LPS are also an important virulence factor of *Brucella* that are different and non-classical and are less toxic and less active than the LPS isolated from *E. coli* [17]. Lipid A in the LPS of *Brucella* is structurally distinct from that of other Gram-negative bacteria in that the base component is diaminoglucose rather than glucosamine, it has longer acyl groups, and it is connected to the core by amide bonds rather than ester and amide bonds [18]. The LPS of *Brucella* strains with rough colonies are structurally similar to the LPS of *Brucella* strains with smooth colonies, but the O-antigen is reduced or absent [19]. *Brucella* can enter cells by connecting to lipid rafts on the cell surface through the O-antigen. The apoptosis of host cells can be inhibited by *Brucella* strains through the interaction between the O-antigen of S-LPS and TNF-α. Thus, infected cells that die do not release specific factors to activate the immune system and evade host immune monitoring [20]. 

*Brucella suis* S2 is the most widely used animal vaccine against brucellosis in China. Some studies have confirmed that *Brucella suis* S2 is pathogenic to humans and should not be used for inoculation of pregnant animals [21]. Studies have reported that *B. melitensis* LPS not only increase the levels of interleukin (IL-1β and IL-6) and antibodies (IgM and IgG), but also decrease the levels of P4, E2, and testosterone without causing systemic dysfunction [22]. However, the effects and possible mechanisms of *Brucella* LPS-caused pregnancy loss remain to be revealed.

Therefore, this research mainly investigated the role of *Brucella suis* S2 LPS in endometrial decidualization by histologic observation and detecting the expression of decidualization-related factors. This was combined with the stromal cell-induced decidual model in vitro to explore the role of *Brucella suis* S2 LPS during the decidualization process of pregnant mice and the effect of LPS in early pregnancy abortion in the mouse. Our study will provide a basis for exploring new anti-*Brucella* preparations and exploring new targets for brucellosis prevention and control. 

## 2. Results

### 2.1. Identification of Brucella LPS 

**Figure 1:**
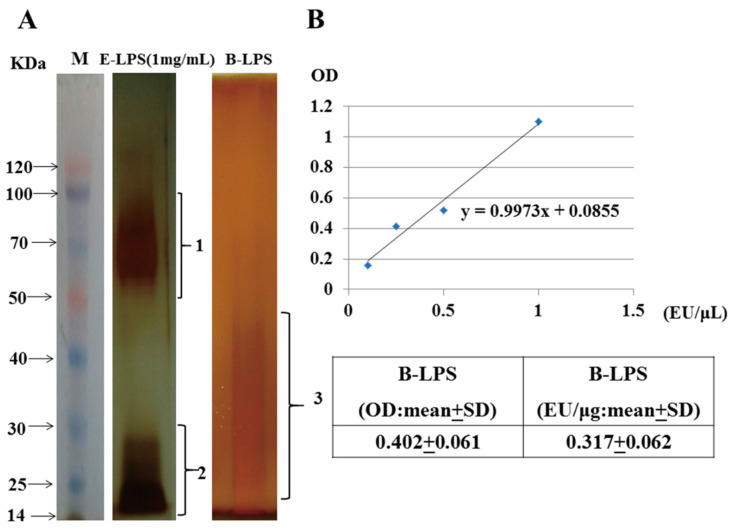
Extraction and characterization of lipopolysaccharides from *Brucella suis* vaccine strain 2 (S2). (**A**) *Brucella suis* vaccine strain 2 (*Brucella suis* S2) was isolated by TSA and amplified by TSB. The structure distribution of *Brucella suis* S2 LPS (1 × 10^9^ CFU/mL) extracted with a bacterial lipopolysaccharide extraction kit was detected with the sodium dodecyl sulfate-polyacrylamide gel electrophoresis (SDS-PAGE) silver staining method using 1 mg/mL of commercial *E. coli* LPS as a control. M: Marker, E-LPS: *E. coli* lipopolysaccharide, B-LPS: *Brucella suis* S2 lipopolysaccharide, 1 refers to the O-antigen, 2 and 3 refer to the lipid A core. (**B**) Lipopolysaccharide activity of *Brucella suis* S2 was detected with a Limulus amebocyte lysate kit. The data represent the mean ± standard deviations from three independent experiments.

*Brucella* LPS have low immunogenicity and are one of the main virulence factors used by *Brucella* to evade host immunity. Therefore, we first analyzed the differences between *Brucella* LPS and *E. coli* LPS structurally. The results of silver staining showed that the commercial *E. coli* LPS were mainly distributed in the range of 50–100 KDa (the O-antigen) and 14–30 KDa (the lipid A core). The dispersive distribution of *Brucella suis* S2 LPS was in the size range of 20–50 KDa (the lipid A core) (Figure 1A). The structure of *E. coli* LPS contains the O-antigen and lipid A core. In contrast, the structure of LPS derived from *Brucella suis* S2 only has the lipid A core. The Limulus amebocyte lysate activity test showed that the activity of 1 μL (1 ng) of *Brucella suis* S2 LPS was 0.317 ± 0.062 EU/μL (Figure 1B). 

### 2.2. Brucella LPS Impairs Embryo Implantation in Mice 

**Figure 2:**
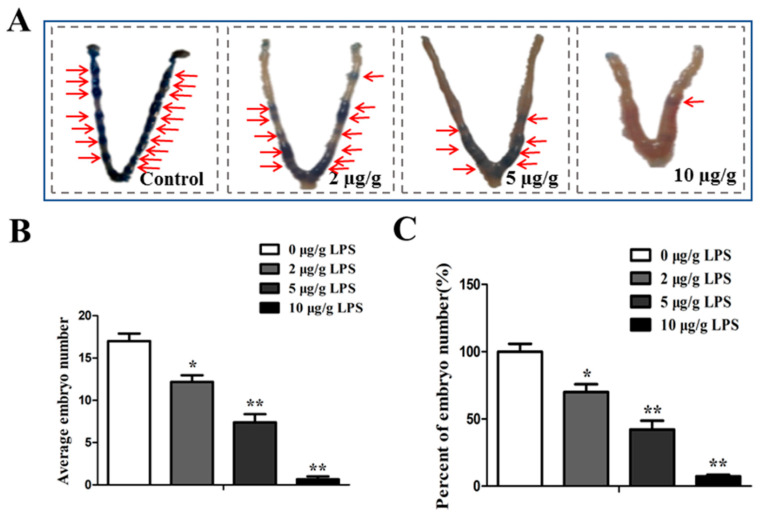
Effects of different concentrations of *Brucella* LPS on mouse embryo implantation. (**A**) The pregnant mice were intraperitoneally injected with different doses of *Brucella suis* S2 LPS (0, 2, 5, or 10 µg/g) on the 4th dpc, and 0.3 mL 1% trypan blue was injected into the tail vein on the 6th dpc. The mice were killed by cervical dislocation 10 min later and embryo implantation in the mouse uterus was observed. The red arrow indicates the implantation site. (**B**) The average number of implanted embryos in each group was calculated, and the average number of implanted embryos = total number of implanted embryos/number of pregnant mice. (**C**) Calculation of the relative implantation rate (%). The values represent the mean ± standard deviations for three duplicates (* *p* < 0.05; ** *p* < 0.01 compared to the control).

As shown in Figure 2A, different concentrations of *Brucella suis* S2 LPS impaired the ability of embryos to implant in pregnant mice on the 4th dpc in a dose-dependent manner. In this study, 10 µg/g of LPS treatment induced almost 100% blastocyst loss in pregnant mice (Figure 2B,C, *p* < 0.01), 5 µg/g of LPS treatment induced about 60% blastocyst loss in pregnant mice (Figure 2B,C, *p* < 0.01), and 2 µg/g of LPS treatment induced about 20% blastocyst loss in pregnant mice (Figure 2B,C, *p* < 0.05). 

### 2.3. Brucella LPS Treatment Impairs the Histological Morphology of the Uterus in Pregnant Mice

**Figure 3:**
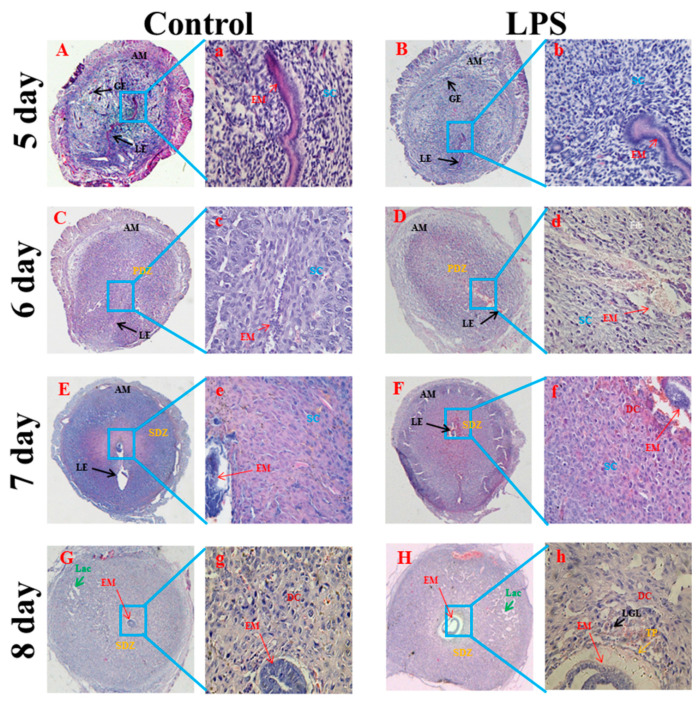
Effect of *Brucella* LPS on endometrial cell structure in pregnant mice. The pregnant mice were intraperitoneally injected with 5 µg/g of *Brucella suis* S2 LPS on the 4th dpc, and the uterine tissues of the mice were collected on the 5th–8th dpc and observed by HE staining ((**A**–**H**) (40×) and (**a**–**h**) (200×)). AM: contralateral mesangium; EM: embryo site; LE: luminal epithelium; GE: glandular epithelium; SC: stromal cell; PDZ: primary decidual area; SDZ: secondary decidual area; DC: decidual cells; Fib: fibrinolysis; Lac: abundant decidual space; TP: trophoblast; LGL: large granular lymphocyte.

Mice on the 5th dpc had a large number of uterine glands, large gaps, gland growth and curvature, and endometrial stromal cell hypertrophy (Figure 3A). The tissue morphology of the uterus of pregnant mice treated with *Brucella suis* S2 LPS did not change significantly on the 5th dpc (Figure 3B). On the 6th dpc, endometrial blood vessels proliferated, the number of uterine glands decreased, stromal cells gradually differentiated into decidual cells, and the primary decidual zone was formed (Figure 3C). However, fibrinolysis was observed in decidualized regions of the *Brucella suis* S2 LPS treated group (Figure 3D). On the 7th–8th dpc, the uterine cavity gradually closed, and the developing embryo and trophoblast tissues were visible. Endometrial stromal cells were fully proliferated and differentiated into decidual cells, the uterine glands were gradually reduced, and the blood vessels became more abundant (Figure 3E,G). In contrast, the decidual cells near the embryo were permeated by large granular cells. There were a large number of gaps in the decidual area, and cell necrosis occurred around the embryo in the *Brucella suis* LPS treatment group (Figure 3F,H). 

### 2.4. Brucella LPS Decreases the Expression of Decidual-Related Factors in Pregnant Mice

**Figure 4:**
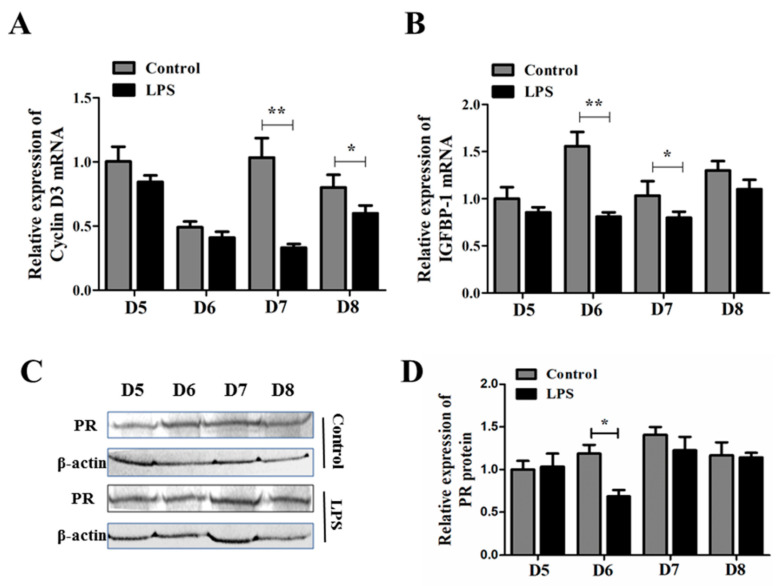
Effect of *Brucella* LPS on the expression of decidual-related factors in pregnant mice. (**A**,**B**) Expression of Cyclin D3 and IGFBP-1 mRNA in the uterus of pregnant mice on the 5th–8th dpc with or without *Brucella suis* S2 LPS treatment. The data represent the mean ± standard deviations from three independent experiments (* *p* < 0.05; ** *p* < 0.01). (**C**) Expression of the PR protein in the uterus of pregnant mice on the 5th–8th dpc with or without *Brucella suis* S2 LPS treatment. The data represent three independent experiments. (**D**) Band intensity measurement of the PR for three independent results was determined by densitometry. The data represent the mean ± standard deviations from three independent experiments (* *p* < 0.05).

The decidual tissue formed by the proliferation and redifferentiation of endometrial stroma stimulated by decidualization inducers is essential for the establishment and maintenance of pregnancy. Hence, we determined whether the transcription of decidualization-related factors is affected by *Brucella suis* S2 LPS. The expression levels of Cyclin D3 and IGFBP-1 mRNA on the 5th–8th dpc of pregnant mouse uterus treated with *Brucella suis* S2 LPS or PBS were analyzed by the qRT-PCR method. In this study, the levels of Cyclin D3 in LPS-treated mouse uterus were significantly decreased in comparison with that in PBS-treated mouse uterus on the 7th dpc (Figure 4A, *p* < 0.01) and the 8th dpc (Figure 4A, *p* < 0.05). At the same time, the mRNA levels of IGFBP-1 in the LPS-treated mouse uterus were decreased in comparison with that in the PBS-treated mouse uterus on the 6th and 7th dpc (Figure 4B, *p* < 0.05). Progesterone receptor (PR) protein expression is important for successful embryo implantation. Hence, we also detected the protein expression of the PR by the pregnant mouse uterus treated with *Brucella suis* S2 LPS or PBS on the 5th–8th dpc by the western blot method. According to our results, the protein level of the PR in the LPS-treated mouse uterus was decreased in comparison with that in the PBS-treated mouse uterus only on the 6th dpc (Figure 4C,D, *p* < 0.05).

### 2.5. Brucella LPS Inhibited the Decidualization of Murine Endometrial Stromal Cells Induced by Estradiol (E2) and Progesterone (P4)

**Figure 5:**
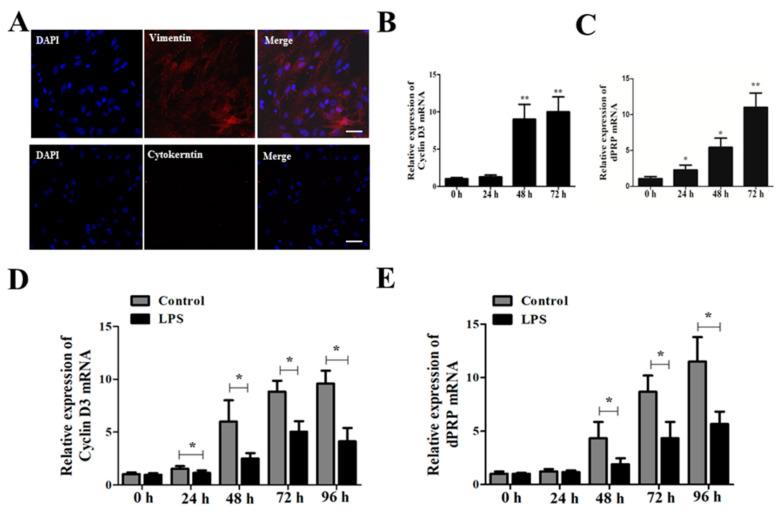
Effects of Brucella LPS on artificially induced decidualization of murine endometrial stromal cells. (**A**) Isolation and identification of murine endometrial stromal cells (ESCs). Immunofluorescent detection of Vimentin and Cytokeratin in ESCs at Passage 3; bar = 50 μm. (**B**,**C**) Estradiol 10 nmol/L (E2) and progesterone 1 μmol/L (P4) were used to induce decidualization of ESCs in vitro. ESCs were harvested after 24 h, 48 h, and 72 h of treatment. The mRNA expression levels of decidualization marker molecules Cyclin D3 and dPRP in mouse endometrium were detected by qRT-PCR. The data represent the mean ± standard deviations from three independent experiments (* *p* < 0.05; ** *p* < 0.01). (**D**,**E**) ESCs were treated with 10 nmol/L E2 and 1 μmol/L P4 for 24 h, and then treated with 1 mg/mL Brucella Suis S2 LPS and cultured for 24 h, 48 h, 72 h, and 96 h. qRT-PCR was used to detect the mRNA expression of decidual marker molecules Cyclin D3 and dPRP in mouse endometrium. The data represent the mean ± standard deviations from three independent experiments (* *p* < 0.05).

In this study, ESCs derived from the uteruses of mice were first isolated and purified. All ESCs at Passage 3 stained positive for the stromal cell marker Vimentin and stained negative for Cytokeratin (Figure 5A). Induction of ESCs for 24 h by estradiol (E2) and progesterone (P4) significantly induced the mRNA expression of decidualization marker molecules Cyclin D3 and dPRP in a dose-dependent manner (Figure 5B,C, *p* < 0.05). However, *Brucella suis* S2 LPS treatment significantly inhibited the expression of decidualization marker molecules Cyclin D3 and dPRP mRNA starting at 24 h (Figure 5D,E, *p* < 0.05).

## 3. Discussion

*Brucella* is a facultative intracellular bacterium that harms the reproductive systems of humans and animals, causing preterm birth, abortion, placenta retention, weak fetus, malformed fetus, and stillbirth [1]. Vaccination remains the main means of combating brucellosis. However, existing animal vaccines can cause abortion and are highly toxic to humans [23]. 

*Brucella* is divided into smooth (S) and rough (rough) types according to whether the LPS on the outer membrane contain the O-chain structure [24,25]. The O-chain is the antigenic determinant of *Brucella*, which is a repeated structure of oligosaccharides composed of three to five glycogroups. The composition and structure of the glycogroups are different in different strains and different groups of the same species, thus determining their species and type specificity [26]. The apoptosis of host cells can be inhibited by LPS derived from *Brucella* strains with smooth colonies through the interaction of the O-antigen with TNF-α. Through the same mechanism, dead cells do not release certain factors that activate the immune system, and thus, they evade host immune surveillance [20]. 

The structure of LPS derived from *Brucella* strains with rough colonies is similar to that of LPS derived from *Brucella* strains with smooth colonies, except that the O-antigen is reduced or absent [19]. *B. suis* belongs to the smooth colony type of *Brucella*. However, in our study, LPS derived from the *B. suis* S2 vaccine strain was found to lack the O-antigen, and it changed from smooth LPS to rough LPS. In studies of *Bacillus canis* and *Bacillus ovis*, it was found that a lack of the O-antigen enhanced their lethality in natural hosts [27]. In a study of *Brucella* pathogenicity, it was found that the release of TNF-α and immune stimulation in mice were strengthened during the process of rough colony *Brucella* infection compared with those during the process of smooth colony *Brucella* infection [28]. Therefore, the lack of the LPS O-antigen in a rough colony of *Brucella* can induce a stronger response and participate in the injury process, such as abortion. 

Different sources of LPS trigger pathological processes associated with various diseases. Some studies have confirmed that LPS derived from *E. coli* or *Porphyromonas gingivalis* can cause fetal weight loss and malformations and embryonic lethal effects [28]. In another study, *Salmonella enteritidis* LPS also increased the miscarriage rate in pregnant mice from 15.5% to 42.0% [29]. In this study, it was found that the structure of LPS derived from *Brucella suis* S2 was significantly different from *E. coli* LPS. The structure of the *E. coli* LPS contains the O-antigen and a lipid A core. In contrast, the structure of LPS derived from *Brucella suis* only has a lipid A core. It was confirmed that *Brucella* LPS showed heterogeneity not only at the level of the O polysaccharides but also at the level of the core oligosaccharides and lipid A. Regardless of the heterogeneity of lipid A, the basic structure of the lipid A skeleton remains unchanged [30]. Studies have found that *Brucella* lipid A is only related to the production of IL-10, and it has little effect on the induction of pro-inflammatory cytokines [26]. It is also possible to understand why *Brucella* LPS fails to stimulate Flt3 dendritic cells to produce IL-12p70 and IFN-γ to support the proliferation of CD4 or CD8 T cells in vitro [31].

The decidua is a maternal–fetal interface formed by the maternal tissue of the endometrium implanted with blastocysts under the action of ovarian steroid hormones and the progeny trophoblast layer [4]. Decidualization of uterine stromal cells is necessary for normal embryo implantation, normal pregnancy, and initiation of labor, and it is an important step in a successful pregnancy [32]. LPS administered on the 7th dpc induce 100% resorption of the blastocysts with no systemic effects in the mother, showing that the decidua, trophoblast, and embryo are highly sensitive to pro-inflammatory molecules such as LPS [33,34]. LPS disrupt the maternal immune balance by inducing the release of inflammatory cytokines and subtle immune responses at the maternal–fetal interface during embryo implantation, which may be one of the causes of embryo implantation failure or abortion [35]. In our study, we also found that *Brucella* LPS caused early embryo loss of pregnant mice in a dose-dependent manner. *Brucella* LPS treatment resulted in fibrinolysis in the decidual area of the uterus on the 6th dpc, infiltration of large granular cells among the decidual cells near the embryo in the uterus on the 8th dpc, a large number of gaps in the decidual area, and cell necrosis around the embryo. These results suggest that *Brucella* LPS affect the histological morphology of the decidua in early pregnancy.

On the 5th dpc, stromal cells in the proliferative stage around the implantation blastocyst can express a large amount of Cyclin D3, which is decreased in the primary decidual region during the stopping proliferation stage, and it has strong expression in the secondary decidual region [36]. The expression of Cyclin D3 was significantly inhibited by *Brucella* LPS treatment on the 7th and 8th dpc. IGFBP-1 is considered to be one of the most common marker molecules during decidualization in humans and animals [37]. In our study, it was also found that *Brucella* LPS treatment significantly inhibited the expression of IGFBP-1 in the uterus of mice on the 6th and 7th dpc. In addition, P4 can cause decidualization of endometrium through the progesterone receptor (PR) [38]. *Brucella* LPS treatment inhibited the protein expression of the PR on the 7th and 8th dpc. 

In order to further explore the effect of *Brucella* LPS on decidualization in mice, endometrial stromal cells were isolated and successfully induced by 1 μmol/L P4 and 10 nmol/L E2 to establish a decidualization model of endometrial stromal cells in vitro. The expression of Cyclin D3 and decidualization prolactin-associated protein (dPRP) were also inhibited by LPS treatment of endometrial stromal cells in vitro. It is important to note that research strategies and the pharmaceutical industry should take into account the different biological activities of LPS, depending on the source of the bacteria and the long-term consequences of using different doses of LPS. 

## 4. Conclusions

In summary, *Brucella* LPS affect the process of endometrial decidualization in pregnant mice by affecting the structure of the decidua and the expression of decidual marker factors in endometrial stromal cells.

## 5. Materials and Methods

### 5.1. Extraction and Identification of Brucella suis S2 LPS

Lipopolysaccharides (LPS) were extracted from *Brucella suis* S2 with a lipopolysaccharide extraction kit (iNtRON, Lot: 17141, Suwon, Gyeonggi Province, Republic of Korea). Then, the SDS-polyacrylamide gel (SDS-PAGE) silver staining method was used to identify the structure of *Brucella suis* S2 LPS. The specific steps were as follows: a 12% separation gel and a 5% concentration gel were prepared. Then, 10 μL aqueous samples of *Escherichia coli* LPS (*Escherichia coli* 0111:B4, Sigma Chemicals Co., St. Louis, MO, USA) and extracted *Brucella suis* S2 LPS were mixed with an equal volume of 0.1 mol/L Tris-HCl in a water bath at 100 °C and cooled for 5 min, then the 20 μL sample was loaded on the gel. Electrophoresis was performed until the bromophenol blue had moved to the bottom of the separation rubber, and the gel was stripped for silver staining. The specific steps are as follows: 30% ethanol, 10% glacial acetic acid, and 7 g/L periodate to fix the gel at 22 °C for 20 min. Then, the gel is washed 3 times with ddH_2_O, 5 min each time. The gel is then stained with 1 g/L AgNO_3_ for 30 min at 30 °C and washed with ddH_2_O once for 10 s each. Then, it is placed in 30 g/L Na_2_CO_3_(4 °C) in 0.02% formaldehyde for 20 min, and 10% glacial acetic acid is used to stop the color development. The gel is washed with ddH_2_O and photographed.

Limulus amebocyte lysate test for activity: 0.1 mL limulus lysate reagent was added into the test tube without a heat source, 0.1 mL LPS sample was added, and the samples were incubated at 37 °C for 16 min. The OD value was detected by a spectrophotometer at 545 nm wavelength, and the LPS activity was calculated according to a standard endotoxin curve. All procedures involving live *B. suis* S2 cells were performed in a biosafety level 2 facility.

### 5.2. Animal Handling and Grouping

Eight-week-old Kunbai mice were group-housed under a 12 h light:12 h dark cycle (from 8:00 a.m. to 8:00 p.m.) and specific pathogen-free conditions. The temperature of the feeding room was controlled from 20 °C to 22 °C, and the animals were allowed to drink freely. Female mice with pale pink vulvas were selected at 16:00 on the day of superovulation. Each mouse was intraperitoneally injected with 10 IU pregnant mare serum gonadotropin (PMSG) for superovulation. After 48 h, 10 IU of human chorionic gonadotropin (hCG) was injected intraperitoneally into each mouse. After the hCG injection, the superovulated female mice were randomly placed into cages with male mice at a ratio of 1:1. Vaginal plug detection was performed at 7:00 a.m. the next day, and it was recorded as the 1st dpc.

Pregnant mice were randomly divided into 4 groups, with 6–8 pregnant mice in each group, and they were injected with 0 (control), 2, 5, or 10 µg/g *Brucella suis* S2 LPS on the 4th dpc. On the 6th dpc, the pregnant mice were killed to observe the status of abortion and to collect the uterine tissue. All of the animal experiments were approved by the ethics committee for animal care and experiments of Northwest A&F University (protocol code: 2019ZX08002035; date of approval: 18 November 2019).

### 5.3. Isolation and Identification of Mouse Endometrial Stromal Cells (ESCs)

The uterus was removed and trimmed to remove the excess adipose mesenchymal tissue. The uterine tissue was surgically cut and transferred to a 4 mL sterile centrifuge tube. The primary cells were separated by the 0.25% trypsin combined with collagenase II method. The isolated ESCs were cultured in cell culture dishes at a density of 2 × 10^5^ cells/mL by adding D-MEM/F-12 (D-MEM/F-12 + 10% FBS + 1000 units/mL penicillin +1 mg/mL streptomycin) medium.

The purity of the ESCs was determined by immunocytochemical staining. ESCs on slides were cultured for 3–4 days and fixed with 4% paraformaldehyde for 30 min. The cells were blocked with FBS for 1 h at 37 °C and washed with PBS without Triton X-100. At 4 °C overnight, primary antibodies against Cytokeratin (Abcam, 1:100 dilution, Cambridge, UK) and Vimentin (Abcam, 1:100 dilution, Cambridge, UK) were added. FITC-labeled sheep anti-mouse secondary antibody working solution was dropped on the slides and incubated at 37 °C for 1 h. After washing in PBS without Triton X-100, the slides were stained with DAPI for 10 min. After washing in PBS without Triton X-100, the slides were sealed with neutral gum and observed.

### 5.4. Artificial Induced Decidualization of Endometrial Stromal Cells 

ESCs were seeded into the complete culture medium of DMEM/F12 at a density of 2 × 10^5^ cells/mL for 24 h. The culture medium was then changed to DMEM/F12 medium without FBS. After 24 h of culture, the culture medium was changed to DMEM/F12 + 10% filtered fetal bovine serum + 1000 unit/mL penicillin + 1 mg/mL streptomycin +10 nmol/L estrogen (E2) + 100 nmol/L progesterone (P4) + 1 mg/mL *Brucella suis* S2 LPS. After 24 h, 48 h, and 72 h of additional culture, the cells were collected, and RNA was extracted. 

### 5.5. Observation and Counting of Embryo Implantation Sites

Pregnant mice were injected with *Brucella* LPS on the 4th dpc, and 0.3 mL 1% trypan blue (Sigma Chemicals Co., St. Louis, MI, USA) was injected through the tail vein on the 6th dpc. After 10 min, the mice were sacrificed. The embryo implantation in the uteruses of the mice was observed, and the number of implanted embryos was recorded. The average number of implanted embryos was calculated according to the following formula: Average number of implanted embryos = total number of implanted embryos/number of pregnant mice. 

### 5.6. Histopathological Analysis of the Uterus

The histopathological analysis of the entire uterus was carried out according to a standard procedure. Small portions of recovered uteruses from pregnant animals of each group were dissected out, freed from fat bodies, and fixed in 4 % paraformaldehyde for 24 h. Tissues were dehydrated in a graded series of ethanol, cleared in xylene, infiltrated, and embedded in paraffin wax at 60 °C. Sections 7 mm thick were mounted onto glass slides precoated with poly-L-lysine solution (Sigma-Aldrich, St. Louis, MI, USA) and incubated overnight at 37 °C. The tissue sections were stained with hematoxylin and eosin (HE) solutions and then observed under a microscope (BA400, Motic, Wetzlar, Germany). 

### 5.7. Reverse-Transcriptase Quantitative Polymerase Chain Reaction

Total RNA was isolated from mouse uteruses and ESCs using the TRIzol reagent according to the manufacturer’s recommendations (Invitrogen, Carlsbad, CA, USA). Extracted RNA was dissolved in diethypyrocarbonate-treated water, and the RNA concentration and purity were estimated by reading the absorbance at 260 and 280 nm on a spectrophotometer (Eppendorf, Hamburg, Germany). The ratio of absorption (260 to 280 nm) of all preparations was 1.8 to 2.0. The cDNA samples were synthesized using a PrimeScript RT reagent kit (Takara Bio, Dalian, China) according to the manufacturer’s instructions. Reverse transcriptase-quantitative polymerase chain reaction (qRT-PCR) was performed using the Light Cycler system (iQ5; Bio-Rad Laboratories, Hercules, CA, USA) using a SYBR Premix Ex Taq II Kit (Takara Bio, Dalian, China), according to the manufacturer’s instructions. Each PCR (total volume of 20 µL) consisted of 2 µL reverse transcription product, 0.8 µL of each 10 µm forward and reverse primer, 10 µL SYBR Premix Ex Taq II, and 6.4 µL RNAse-free water. The sequences for the specific primers are given in Table 1. The mRNA quantifications were performed by the 2^−∆∆Ct^ method, and the number of transcripts in each sample was normalized using β-actin as an internal control to correct for differences in cDNA.

### 5.8. Western Blot Analysis

The tissues were weighed, and 200 µL of RIPA buffer (Beijing Solarbio Science & Technology, Beijing, China) was added per 1 g of tissue, followed by homogenization and centrifugation at 14,200× *g* for 5 min. Protein concentration determination was performed using a BCA assay (Nanjing Keygen Biotech, Nanjing, China). Next, 20 µg of total protein per sample was separated by 12% sodium dodecyl sulfate-polyacrylamide gel electrophoresis and electro-transferred to a polyvinylidene fluoride membrane (Millipore, Bedford, MA, USA). After incubation in blocking buffer (10% defatted milk powder in Tris-buffered saline containing 0.1% Tween 20) for 1 h at room temperature, the membrane was incubated overnight with mouse anti-progesterone receptor (PR) monoclonal antibody (10 µg/mL, Santa Cruz Biotechnology, Dallas, TX, USA) or anti-β-actin antibody (1:1000; Beijing CWBIO, Beijing, China) as a loading control at 4 °C. After washing, the membranes were incubated with secondary antibody conjugated to horseradish peroxidase (1:5000; Zhongshan Goldenbridge Biotechnology, Nanjing, China) at room temperature for 1 h. Finally, the immunoreactive bands were visualized using a Gel Image System (Tannon Biotech, Shanghai, China). 

### 5.9. Statistical Analyses

All experiments were replicated at least three times for each group, and the data are presented as the mean ± SEM. The data were analyzed with ANOVA, followed by Fisher’s least significant different test and independent samples Student’s *t*-test, with SPSS software, version 13.0 (SPSS, Chicago, IL, USA). Differences were considered significant at *p* < 0.05.

## Figures and Tables

**Table 1:** The sequences for the specific primers.

Gene	Product Size	Primer Sequence
CyclinD3	138 bp	Sense 5′-TGGATCGCTACCTGTCCTG-3′ Anti-sense 5′-CCTGGTCCGTATAGATGCAAAG-3′
IGFBP-1	139 bp	Sense 5′-CTGCCAAACTGCAACAAGAATG -3′ Anti-sense 5′-GGTCCCCTCTAGTCTCCAGA-3′
dPRP	101 bp	Sense 5′-GGGGTGGAGGTCGTACAAG-3′ Anti-sense 5′-GCGAGTAGAATGACAGCTCCTT-3′
β-actin	228 bp	Sense 5′-AGCCATGTACGTAGCCAT-3′ Anti-sense5′-CTCTCAGCTGTGGTGGTGAA-3′

## Data Availability

The datasets used and analyzed during the current study are available from the corresponding author upon reasonable request.
